# Nrf2 is the key to chemotherapy resistance in MCF7 breast cancer cells under hypoxia

**DOI:** 10.18632/oncotarget.7406

**Published:** 2016-02-15

**Authors:** Jhih-Pu Syu, Jen-Tsan Chi, Hsiu-Ni Kung

**Affiliations:** ^1^ Department of Anatomy and Cell Biology, College of Medicine, National Taiwan University, Taipei, Taiwan; ^2^ Center for Genomic and Computational Biology, Duke University, Durham, NC, USA; ^3^ Department of Molecular Genetics & Microbiology, Duke University, Durham, NC, USA

**Keywords:** Nrf2, hypoxia, drug resistance, antioxidant activity, breast tumor

## Abstract

Hypoxia leads to reactive oxygen species (ROS) imbalance, which is proposed to associate with drug resistance and oncogenesis. Inhibition of enzymes of antioxidant balancing system in tumor cells was shown to reduce chemoresistance under hypoxia. However, the underlying mechanism remains unknown. The key regulator of antioxidant balancing system is nuclear factor erythroid 2-related factor 2 (NFE2L2, Nrf2). In this study, we showed that hypoxia induced ROS production and increased the Nrf2 activity. Nrf2 activation increased levels of its downstream target antioxidant enzymes, including GCLC and GCLM. The Nrf2-overexpressing also confers chemo-resistant MCF7 cells under normoxia. The *in vivo* mouse model also demonstrated that the chemical inhibition of Nrf2 can increase cisplatin (CDDP) cytotoxicity. Together, these results showed that Nrf2 serves as a key regulator in chemotherapeutic resistance under hypoxia through ROS-Nrf2-GCLC-GSH pathway. Therefore, targeting Nrf2 can be a potential treatment for hypoxia-induced drug resistance in breast cancer cells.

## INTRODUCTION

As the solid tumors grow in size, the blood vessels are often defective and cannot supply sufficient oxygen to the tumor tissue, thus producing a low-oxygen microenvironment in a condition called hypoxia. Hypoxia leads to the intracellular oxidative imbalance [1], and also leads to many genetic and metabolic alterations in tumor cells [2-5]. Tumor hypoxia has been shown to increase tumor metastasis [6], and radio- or chemo-therapeutic resistance [7, 8], thus serves as a prognostic marker of poor clinical outcome [9, 10].

Hypoxia also confers resistance to commonly used chemotherapeutic drugs, such as cisplatin (CDDP) [11], carboplatin [8], and doxorubicin [7], in colorectal [12], lung [11], prostate [7], and breast tumors [13]. Many mechanisms have been suggested to be responsible for the hypoxia-induced drug resistance [8]. One prominent potential critical mediator is glutathione (GSH) [14]. Overexpression of glutathione S-transferase (GST) isoenzymes, which catalyzes the conjugation of the GSH to substrates for detoxification, has been implicated in the resistance to chemotherapeutic agents [15–17]. In addition, the inhibition of GSH production with buthionine sulfoximine (BSO) has been shown to inhibit the chemoresistance of tumor cells [18, 19]. These phenomena reveal that antioxidant ability is critically associated with chemoresistance in cancer cells. Therefore, we hypothesized that hypoxia may lead to oxidative stress, and activate antioxidant enzymes to cause hypoxia-induced drug resistance.

The master transcription factor responsible for coordinating antioxidant activity is nuclear factor erythroid 2-related factor 2 (NFE2L2, Nrf2) [20]. Under oxidative stresses, Nrf2 translocates from cytoplasm into nucleus and binds to the antioxidant response element (ARE), which leads to the transcription and translation of phase II antioxidant enzymes, including NAD(P)H quinine oxidoreductase 1 (NQO1), glutamate-cysteine ligase catalytic subunit (GCLC), and glutamate-cysteine ligase modifier subunit (GCLM) [21]. GCLC and GCLM can bind to each other as a heterodimer to promote the synthesis of GSH [22], which is responsible for the intracellular redox homeostasis and chemical detoxification. It has been reported that the inhibition of Nrf2 can sensitize some lung carcinoma cells to chemotherapeutic drugs under normoxic culture conditions [23], but the role of Nrf2 in hypoxia-induced chemotherapeutic resistance in tumors is not well understood. Here, we investigate the role of Nrf2 in hypoxia-induced drug resistance and find Nrf2 is the key regulator for hypoxia-induced drug resistance, and targeting Nrf2 can facilitate the treatment efficiency of cisplatin.

## RESULTS

### Higher drug resistance in MCF7 cells under hypoxia

**Figure 1:**
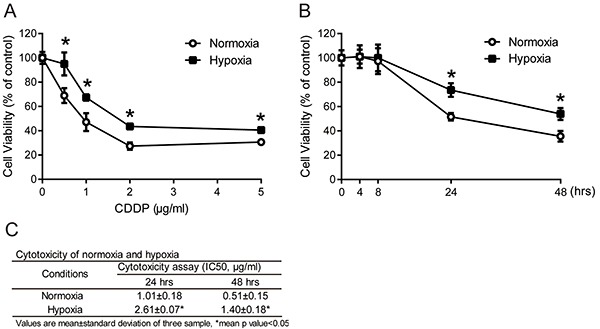
Drug resistance in tumor cells under hypoxia **A.** MCF7 cells were treated with 0.5, 1, 2 and 5 μg/ml cisplatin (CDDP) for 24 hours under normoxia or hypoxia (2% O_2_). N=3, *, P < 0.05 compared with the normoxia. **B.** MCF7 cells were treated with 1 μg/ml CDDP for the indicated times under normoxia or hypoxia. Cell viabilities were measured by MTT assay. N=3, *, P < 0.05 compared with the normoxia. **C.** The IC_50_ value of CDDP on MCF7 cells under normoxia and hypoxia at 24 and 48 hours. Values are mean ±SD of three samples. *, P < 0.05 compared to normoxia.

Solid tumors are usually more resistant to chemotherapy and are associated with a poor prognosis in hypoxic microenvironments [9, 10]. To demonstrate the role of hypoxia in drug resistance, the hypoxic microenvironment was mimicked by a hypoxic chamber with a 2% O_2_ atmosphere. To examine the drug resistance of tumor cells under hypoxia, MCF7 cells were exposed to a common clinical chemotherapeutic medicine, cisplatin (CDDP), in normoxic (20% O_2_) or hypoxic (2% O_2_) conditions. Cell viability was measured by MTT assay. The cell viabilities in MCF7 cells increased significantly under hypoxia compared with normoxia after 24 hours of treatment at all concentrations of CDDP (Figure 1A). To further understand the time-dependent cytotoxicity of CDDP on cells, cells were treated with 1 μg/ml CDDP, and cell viability was measured at different time points between 4 and 48 hours. The cell viabilities with CDDP treatment under hypoxia were significantly increased at 24 and 48 hours compared to normoxia (Figure 1B). The IC_50_ value of MCF7 cells was significantly increased under hypoxia compared to normoxia at 24 (hypoxia: 2.61±0.07 μg/ml, normoxia: 1.01±0.18 μg/ml) and 48 hours (hypoxia: 1.40±0.18 μg/ml, normoxia: 0.51±0.15 μg/ml) (Figure 1C). These results showed that the hypoxic microenvironment leads to a higher drug resistance in cancer cells.

### Regulation of GCLC leads to the alteration of drug resistance under hypoxia

**Figure 2:**
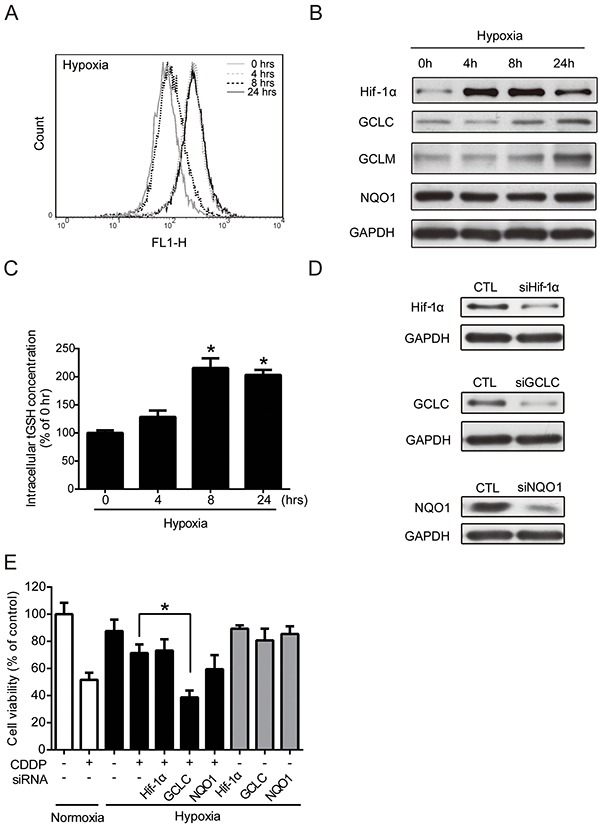
Regulation of antioxidant enzyme activities leads to the alteration of drug resistance under hypoxia **A-D.** MCF7 cells were exposed to hypoxia for 0, 4, 8, 24 hours. (A) The intracellular ROS levels were detected by flow cytometry with DCFHDA staining. (B) The total proteins were extracted from the cells and the Hif-1α, NQO1, GCLC, GCLM, GAPDH levels were detected by western blot. (C) The intracellular total GSH (tGSH) activities were measured by glutathione assay. N=3, *, P < 0.05 compared with the 0 hour group. (D, E) MCF7 cells were transfected with negative control siRNA (siCTL) or siRNAs targeting Hif-1α, GCLC, or NQO1. (D) The effects of siRNA knockdown were examined by western blotting. **E.** After transfection, the cells were treated with 1 μg/ml CDDP for 24 hours under hypoxia. The cell viabilities were examined by MTT assay. N=3, *, P < 0.05 compared to CDDP under hypoxia.

Previous reports have demonstrated that hypoxia can increase intracellular ROS level [24–26]. ROS are toxic but also function as signaling transduction molecules [27]. Hypoxia-induced ROS, particularly that of H_2_O_2_, has been reported to be involved in the regulation of hypoxic responses [28]. However, the physiological contexts and phenotypes of hypoxia-increased ROS are not fully understood. Therefore, the intracellular ROS levels were determined at 0, 4, 8, and 24 hours under hypoxia by flow cytometry with DCFHDA staining. Compared to the control cells at 0 hour, the ROS level increased at 4 hours, decreased at 8 hours, and then increased again at 24 hours under hypoxia (Figure 2A). The change in ROS levels suggested that hypoxia altered the antioxidant ability. Therefore, the protein levels of Hif-1α and several major phase II antioxidant enzymes (NQO1, GCLC, and GCLM) under hypoxia were measured by western blotting. The Hif-1α protein level was significantly increased, which was indicative of hypoxic conditions. The GCLC and GCLM protein levels increased at 8 and 24 hours, whereas the NQO1 level did not change under hypoxia (Figure 2B). The GCLC level reached significance at 8 and 24 hours, and the significance of GCLM was shown at 24 hours (Supplementary Figure S1A). GCLC and GCLM proteins together assemble an enzyme, glutamate cysteine ligase (GCL), which regulates the GSH synthesis and the capacity of antioxidants [22]. To verify the function of GCLC/GCLM proteins, a GSH assay kit was used to detect the total GSH (tGSH) content. Compared with the 0-hour group, the 4-, 8-, and 24-hour groups exhibited an increased intracellular tGSH level under hypoxia (Figure 2C). In summary, hypoxia-induced GCLC/GCLM led to GSH production to balance the intracellular ROS levels.

The cytotoxic effect of CDDP mainly resulted from the inhibition of DNA replication and the induction of apoptosis through the ROS pathway [29]. Therefore, an increase of antioxidant capacity may lead to CDDP resistance. To further understand whether the increased GCLC activities was correlated to hypoxia-induced drug resistance, cells were transfected with siRNAs specifically targeted to Hif-1α, GCLC, or NQO1, and the drug resistance under normoxic and hypoxic conditions was examined. The knockdown efficiency of siRNAs was measured by western blot (Figure 2D), and the statistic results were shown in Supplementary Figure S1B. Then, the cell viability was tested by MTT assay. The cell survival rate of combination treatment of CDDP and GCLC knockdown, but not the knockdown of other siRNAs, was significantly reduced under hypoxia compared with CDDP alone (Figure 2E). Together, these results indicate that the expression and antioxidant function of GCLC is important in drug resistance under hypoxia.

### Activation of Nrf2 leads to drug resistance under hypoxia

**Figure 3:**
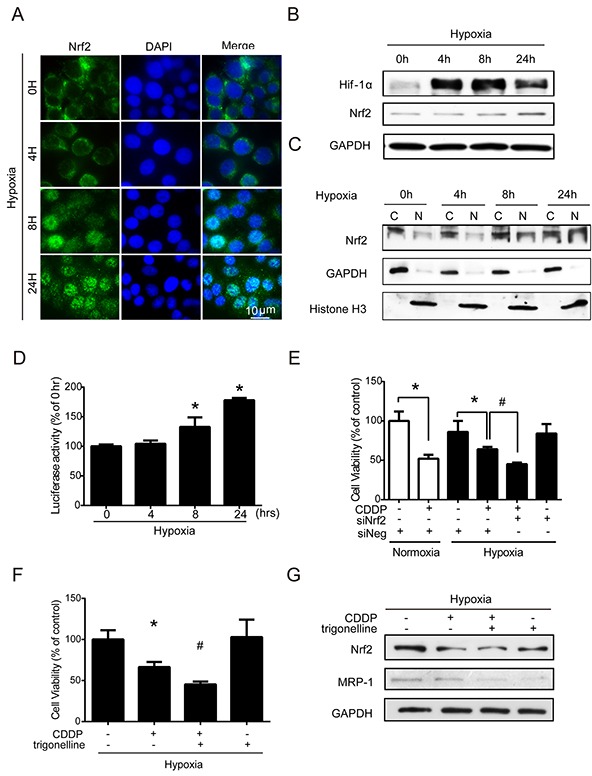
Nuclear translocation of Nrf2 leads to drug resistance under hypoxia **A-C.** MCF7 cells were exposed to hypoxia for 0, 4, 8, and 24 hours. (A) Nrf2 localization was determined by immunocytochemistry (ICC) with an anti-Nrf2 antibody (green fluorescence). Nuclear location was determined by DAPI staining (blue fluorescence). (B, C) Total proteins and cytosolic (C)/nuclear (N) proteins were extracted. The protein levels of Hif-1α, Nrf2, GAPDH, and Histone H3 were detected by western blotting. GAPDH was the loading control for the cytosolic fraction, and Histone H3 was the loading control for the nuclear fraction. **D.** MCF7 cells were transfected with pARE-CMV and pRL-CMV plasmids. After transfection, the cells were exposed to hypoxia for 0, 4, 8, and 24 hours, and the firefly and Renilla luminescence activities were detected by Dual-Glo luciferase assay. The luciferase activity was represented by firefly luminescence normalized with Renilla luminescence. N=3, *, P < 0.05 compared with the 0 hour group. **E.** MCF7 cells were treated with negative siRNA (siNeg) and Nrf2 siRNA for 24 hours under hypoxia, and the cell viability was determined by MTT. N=3, *, P < 0.05 compared with the control. #, P < 0.05 compared with CDDP treatment. **F, G.** MCF7 cells were treated with 1 μg/ml CDDP combined with or without 3 hours of pretreatment with trigonelline (Nrf2 inhibitor) or 1 μM trigonelline alone. (F) The cell viability was determined by MTT. N=3, *, P < 0.05 compared with the control. #, P < 0.05 compared with the CDDP treatment. (G) The total proteins were extracted from the cells and the Nrf2, MRP-1, and GAPDH levels were detected by western blotting.

Nrf2 is a transcription factor and the translocation of Nrf2 from the cytoplasm into the nucleus can increase the transcriptional activities of downstream antioxidant genes [21], including GCLC and GCLM. However, it is unclear whether Nrf2 is involved in the hypoxia-induced drug resistance of cancer cells. To test this possibility, the nuclear translocation of Nrf2 was examined by immunocytochemistry (ICC) and nuclear protein detection assays. The nuclear translocation of Nrf2 (green fluorescence) was dramatically increased at 8 and 24 h. Overlaying the images of Nrf2 and DAPI (cyan fluorescence, used as a nucleus indicator) confirmed the nuclear location of Nrf2 (Figure 3A). The quantitative analysis of cells with Nrf2 positive nucleus were shown in Supplementary Figure S2A, the percentage of Nrf2 positive nucleus increased dramatically from 4 to 24 hours compared to 0 hour under hypoxia. In western blotting, the total Nrf2 expression was significantly increased at 24 h (Figure 3B), and the quantified results of Nrf2 were shown in Supplementary Figure S2B. Hif-1α was used as a hypoxic indicator. Similar results were shown in the nuclear (N)/cytoplasmic (C) fractions, and Nrf2 was significantly increased in the nucleus at 8 and 24 hours. GAPDH and histone H3 were used as the loading controls for cytoplasmic and nuclear fractions (Figure 3C), and the quantified results were shown in Supplementary Figure S2C. The results of ARE luciferase reporter assay also showed a significant increase in Nrf2 activity at 8 and 24 hours under hypoxia (Figure 3D). To further clarify whether the Nrf2 activity was associated with drug resistance under hypoxia, cells were treated with siRNA targeting Nrf2 or with a specific Nrf2 inhibitor, trigonelline [30], to inhibit Nrf2 activity. The inhibition efficiencies of siRNA and trigonelline were first verified, as shown in Supplementary Figures S3A and S3B. Thus, based on the cell viabilities, the combination treatment of CDDP with Nrf2 siRNA and trigonelline significantly enhanced the drug sensitivity of CDDP under hypoxia (Figure 3E, 3F).

In addition, it has been reported that multidrug resistance-associated protein 1 (MRP-1) can efflux the chemotherapeutic drugs out of cells to decrease drug efficacy in cancer cells [31]. Interestingly, we found that the MRP-1 protein level was inhibited by trigonelline under hypoxia (Figure 3G), and the quantified results of MRP-1 were shown in Supplementary Figure S3C. This finding suggested that Nrf2 not only regulates the balance of ROS homeostasis but also may be involved in drug efflux to increase drug resistance under hypoxia. Together, these results indicated that hypoxia-induced Nrf2 activation is very important in drug resistance under hypoxia.

### Nrf2 activation is increased by hypoxia-induced intracellular ROS production

**Figure 4:**
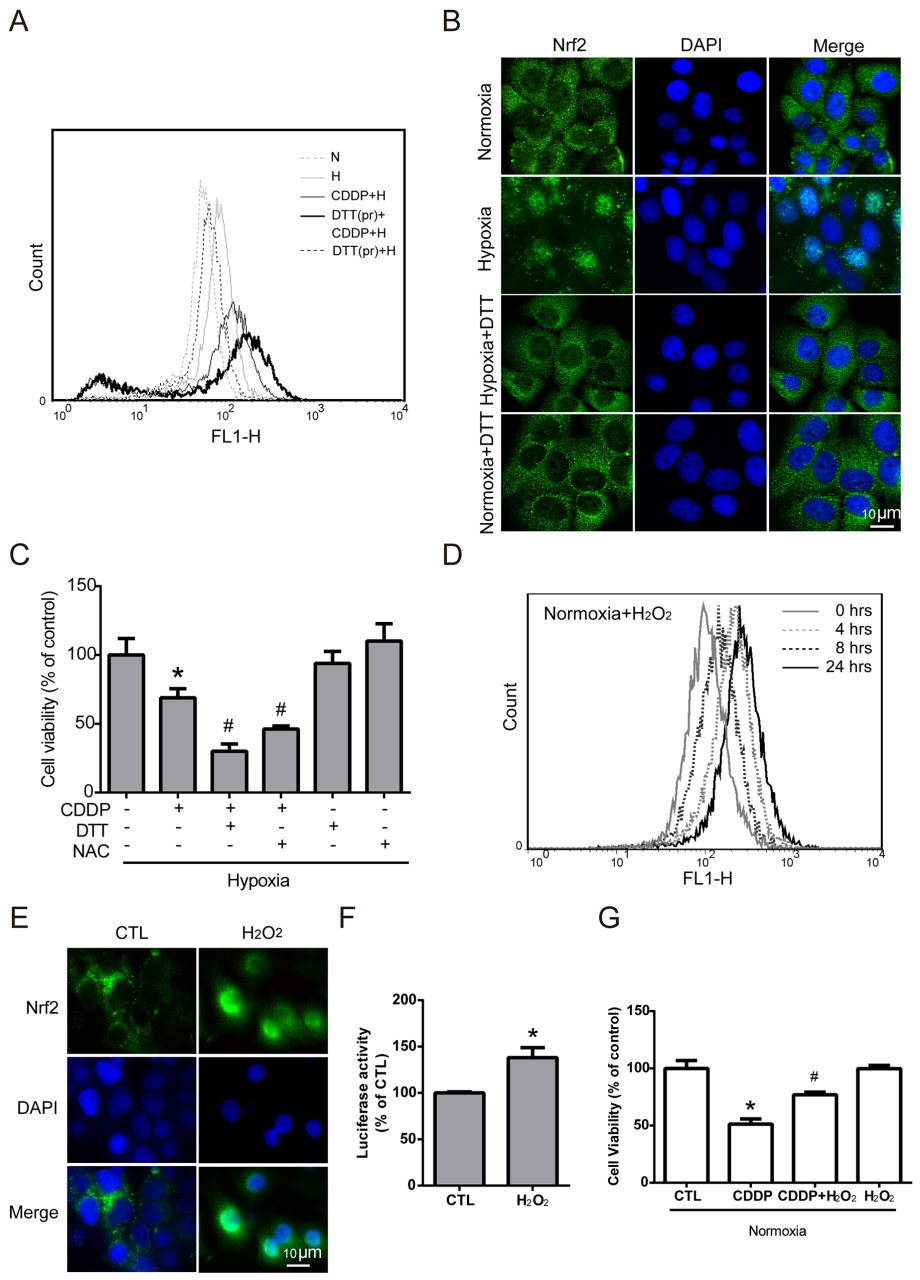
Nrf2 activation is increased by hypoxia-induced intracellular ROS production **A.** MCF7 cells were pretreated with DTT (DTTpr) for 2 hours and followed by combined treatment with CDDP under hypoxia (H) for 24 hours. The intracellular ROS levels were detected by flow cytometry with DCFHDA staining. N indicates normoxic conditions, and H stands for the hypoxia. **B.** Cells were pretreated with DTT for 3 hours under 24 hours of hypoxia, and the localization of Nrf2 was determined by ICC with anti-Nrf2 antibody (green fluorescence). Nucleus was stained by DAPI (blue fluorescence). **C.** Cells were pretreated with DTT or NAC for 3 hours, and CDDP was added to treat cells for 24 hours under hypoxia. The cell viability was measured by MTT. N=3, *, P < 0.05 compared with the control group. #, P < 0.05 compared with the CDDP group. **D.** MCF7 cells were treated with 40 μM H_2_O_2_ for 0-24 hours and the intracellular ROS levels were detected by flow cytometry with DCFHDA staining. **E.** The localization of Nrf2 was determined by ICC with anti-Nrf2 antibody (green fluorescence). The nucleus was stained with DAPI (cyan fluorescence). **F.** The luciferase activity was measured by Dual-Glo luciferase assay with 24 hours of H_2_O_2_ treatment. N=3, *, P < 0.05 compared with the control group. **G.** Cells were pretreated with H_2_O_2_ for 3 hours, and CDDP was then added to treat cells for 24 hours under normoxia. The cell viability was measured by MTT. N=3, *, P < 0.05 compared with the control group. #, P < 0.05 compared with the CDPP treatment group.

Previous studies have shown that Nrf2 activity is induced by ROS imbalance [32], and many studies have reported ROS imbalance under hypoxia. Therefore, we assumed that ROS imbalance maybe a key modulator of hypoxia-induced Nrf2 activity. To further understand the role of hypoxia-induced ROS in Nrf2 activation, MCF7 cells were pretreated with dithiothreitol (DTT, a ROS scavenger) for 2 hours, and then treated with CDDP under hypoxia for 24 hours. The intracellular ROS level was increased dramatically in the group with the combination of DTT pretreatment and CDDP compared with the CDDP alone group (Figure 4A). This result showed that pretreatment with DTT could inhibit the ROS balance system and lead to greater oxidative stress with CDDP treatment under hypoxia. To confirm the relationship between hypoxia-induced ROS and Nrf2 activity, the translocation of Nrf2 was detected by ICC method. Overlaying the Nrf2 (green fluorescence) and DAPI (cyan fluorescence) in hypoxia group showed the translocation of Nrf2, and the DTT pretreatment can reverse the translocation of Nrf2 (Figure 4B). Then, to demonstrate the relationship between hypoxia-induced ROS and drug resistance, MCF7 cells were treated with a combination of CDDP and pretreatment of DTT or N-acetyl-L-cysteine (NAC, another ROS scavenger) under hypoxia, and the cell viability was significantly decreased compared to the CDDP group (Figure 4C).

To further demonstrate whether ROS is the key regulator of hypoxia-induced Nrf2 activity and drug resistance, MCF7 cells were treated with different concentrations of H_2_O_2_ (0, 20, 40, 80, 100 μM) for 24 hours, and the cell viability showed that the cytotoxic effect of H_2_O_2_ on MCF7 cells was greater while the concentration reached 80 μM (Supplementary Figure S4). Therefore, 40 μM H_2_O_2_ was chosen to activate Nrf2 without causing cell death in the following experiments. MCF7 cells were treated with 40 μM H_2_O_2_ for 0, 4, 8, and 24 hours, and the intracellular ROS levels were detected by flow cytometry with DCFHDA staining. Surprisingly, the ROS level changed under H_2_O_2_ treatment in the normoxia group showed a similar pattern (Figure 4D) to the previous result under hypoxic conditions (Figure 2A): the ROS level increased slightly at 4 hours, decreased at 8 hours, and increased again at 24 hours. This result indicated that the ROS imbalance under hypoxia may be the same as that with 40 μM H_2_O_2_ treatment under normoxia.

Previous studies have indicated that ROS may act as a signaling molecule to activate Nrf2 [32]. As shown in Figure 4E, overlaying Nrf2 (green fluorescence) and DAPI (cyan fluorescence) in the H_2_O_2_ treated group confirmed the nuclear translocation of Nrf2. In addition, Nrf2 activity was significantly increased with H_2_O_2_ treatment, as measured by the luciferase reporter assay (Figure 4F). Furthermore, the cell viability was dramatically increased with combination treatment of CDDP and H_2_O_2_ compared to the CDDP group under normoxia (Figure 4G). Together, these results indicated that Nrf2 activity was increased by hypoxia-induced ROS production.

### Nrf2 contributes to the increase of drug resistance

**Figure 5:**
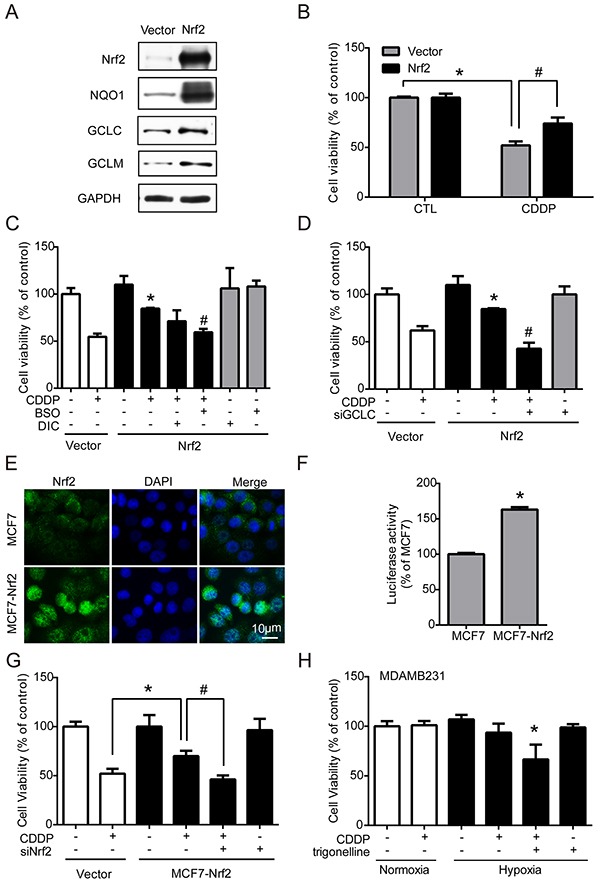
Nrf2 contributes to the increase of drug resistance MCF7 cells were transfected with vector control (vector) and Nrf2 plasmid (Nrf2). **A.** The protein levels of Nrf2, NQO1, GCLC, GCLM and GAPDH were detected by western blotting. **B.** MCF7 cells were treated with 1 μg/ml CDDP for 24 hours, and cell viability was determined with MTT. N=3, *, P < 0.05 compared with the vector control. #, P < 0.05 compared to the vector control with CDDP treatment. **C, D.** The cells were treated with CDDP with one of the following treatments: 3 hours of pretreatment with 100 μM buthionine sulfoximine (BSO), 3 hours of pretreatment with 40 μM dicoumarol (DIC, NQO1 inhibitor), or transfection with GCLC siRNA following cell viability assays. N=3, *, P < 0.05 compared with CDDP treatment in the vector groups. #, P < 0.05 compared to the CDDP treatment in the Nrf2 overexpression groups. **E.** The localization of Nrf2 was determined by ICC with anti-Nrf2 antibody (green fluorescence). The nucleus was stained by DAPI (blue fluorescence). **F.** The luciferase activity was measured by Dual-Glo luciferase assay. N=3, *, P < 0.05 compared with the MCF7 cells. **G.** MCF7-Nrf2 cells were transfected with Nrf2 siRNA and this is followed by 24 hours of CDDP treatment. The cell viability was examined by MTT assay. N=3, *, P < 0.05 compared with the CDDP group in the vector control. #, P < 0.05 compared to the MCF7-Nrf2 with CDDP. **H.** MDAMB231 cells were treated with 1 μg/ml CDDP with or without 3 hours pretreatment of trigonelline. The cell viability was determined by MTT. N=3, *, P < 0.05 compared with CDDP treatment under hypoxia.

Nrf2 is well-known for its role in antioxidant regulation [20], and was responsible for drug resistance in our results. Hypoxia-induced ROS imbalance increased Nrf2 activity and enhanced the cell protection mechanism under chemotherapy. Therefore, transient and stable Nrf2 expression systems were used to verify whether the activation of Nrf2 is the key regulator of drug resistance. First, MCF7 cells were transfected with the vector control (vector) or Nrf2 plasmid (Nrf2). The protein levels of Nrf2 and the downstream phase II enzymes NQO1, GCLC, and GCLM were all significantly increased in the Nrf2 overexpression group (Figure 5A), and the quantified results were shown in Supplementary Figure S5. Then, cells were treated with CDDP for 24 hours, and cell viability was measured by MTT assay. The results showed that the drug resistance was significantly increased in Nrf2-expressing cells (Figure 5B). Furthermore, the Nrf2-expressing cells were treated by CDDP combined with BSO (GCLC inhibitor), Dicoumarol (NQO1 inhibitor) or siGCLC RNA. CDDP treatment combined with BSO (Figure 5C) or siGCLC RNA (Figure 5D) significantly decreased the drug resistance in Nrf2-expressing cells.

Then, a stable Nrf2-overexpressing cell line, MCF7-Nrf2, was established by neomycin selection. MCF7-Nrf2 cells showed more nuclear translocation of Nrf2 than normal MCF7 cells (Figure 5E). In contrast, the protein levels of Nrf2 and GCLC were significantly increased in MCF7-Nrf2 cells; the quantified results were shown in Supplementary Figure S6A. Nrf2 activity was also significantly increased in MCF7-Nrf2 cells, as measured by luciferase reporter assay (Figure 5F). With a cell viability assay, similar results were observed: MCF7-Nrf2 cells were more resistant to CDDP than MCF7 cells (Figure 5G). To confirm whether Nrf2 is the key regulator of drug resistance, cells were transfected with siNrf2 to inhibit the expression of Nrf2 and treated with CDDP for 24 hours. The drug resistance induced by Nrf2 overexpression was blocked by siNrf2 (Figure 5G). Similarly, MDAMB231 cells, which had higher Nrf2 expression (Supplementary Figure S6B), were treated with a combination of CDDP and trigonelline under hypoxia, and the drug sensitivity of CDDP was significantly enhanced under hypoxia (Figure 5H). These results suggest that Nrf2 activity in cancer cells plays an important role in drug resistance.

### The combination treatment of CDDP and trigonelline treated breast cancers efficiently in the *in vivo* mouse model, and the TCGA breast cancer data showed that Nrf2 is an important index of the survival rate of patients

**Figure 6:**
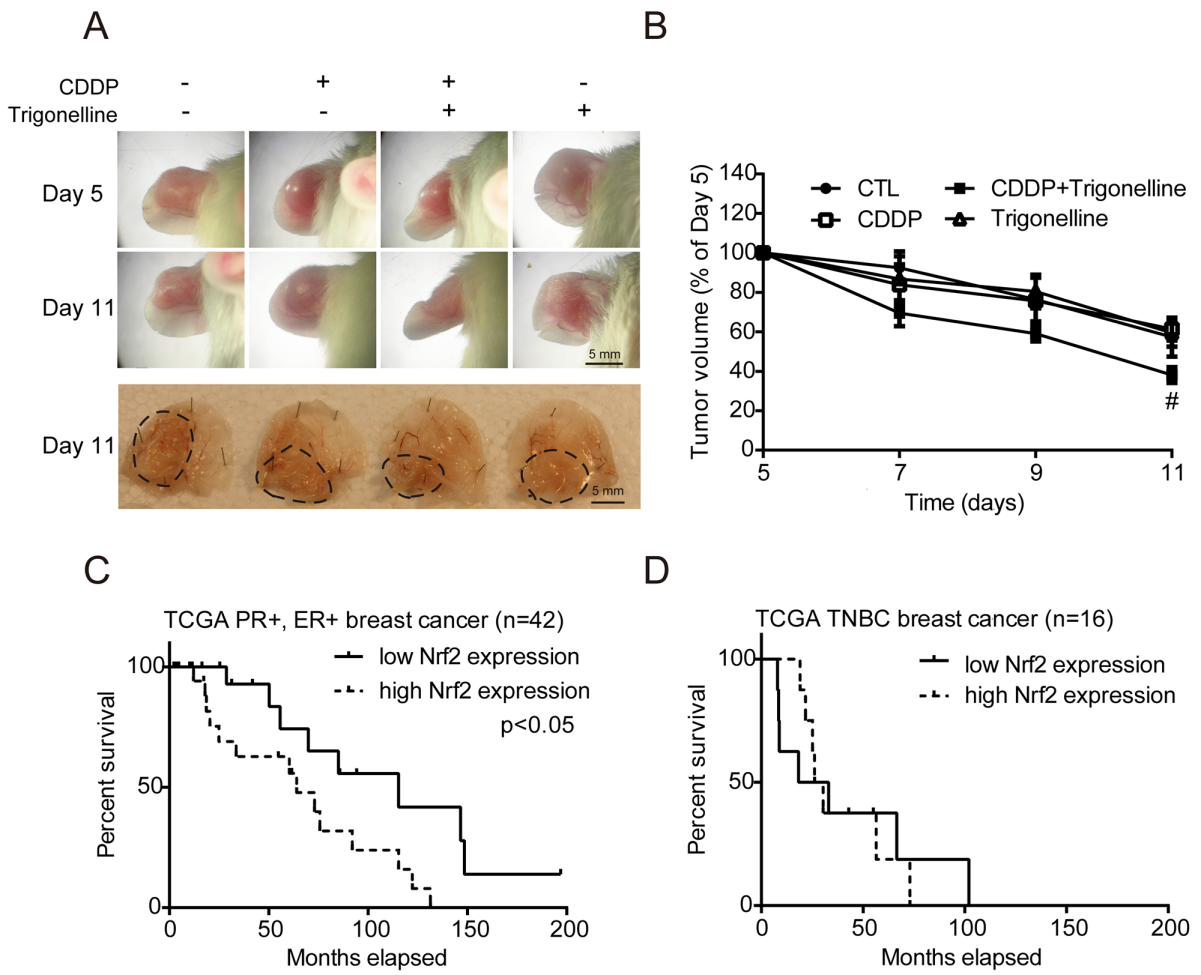
CDDP combined with trigonelline treatment can effectively treat tumors in mice, and the TCGA breast cancer data show the importance of Nrf2 in the survival rate of patients MCF7 cells were injected into the ears of 10-week-old male ICR mice. Mice were randomly separated into four groups, including PBS (CDDP^−^/trigonelline^−^), CDDP, CDDP combined with trigonelline, and trigonelline alone. **A.** Drugs were administered to the tumors on days 5, 7, 9 and 11 after cell injection, and the pictures were taken on day 5 and day 11. Dashed lines indicated the tumor outline (upper panel: whole ear; lower panel: ears without upper surface skin). **B.** The tumor volumes were measured by digital caliper. N=3, #, P < 0.05 compared to the CDDP treatment group. **C, D.** Relapse-free survival data were from the TCGA database. The breast cancer data were classified into the ER^+^/PR^+^ group and TNBC group. The survival data from two groups were used to generate survival curves with separation of high Nrf2 or low Nrf2 expression. The p-values were examined by Mantel-Cox log-rank test.

To determine whether our findings would be relevant in an *in vivo* xenograft model, MCF7 cells were injected into the ears of 10-week-old male ICR mice. Mice were randomly separated into four groups treated with PBS, CDDP, CDDP combined with trigonelline or trigonelline alone. The conditions of tumor growth on days 5 and 11 were photographed (Figure 6A). The mice were sacrificed on day 11, and the tumors were removed for photography. The tumor size of the CDDP and trigonelline combination group was smaller than that of the CDDP alone group (Figure 6A), the third panel, dashed lines). The tumors treated with a combination of CDDP and trigonelline were significantly smaller than those of the PBS or CDDP treatment alone groups on day 11. The tumor volumes were also measured on days 5, 7, 9, and 11 after cell injection, and the volumes in the group treated with a combination of CDDP and trigonelline were less than those of the other groups, reaching significance on day 11 (Figure 6A and 6B). Since the Nrf2 activation can be indicated by the phosphorylation of Nrf2 [33], the Nrf2 activity was confirmed by IHC method with anti-phospho-Nrf2 antibody in tumor sections (Supplementary Figure S7). Results showed that Nrf2 activity (green fluorescence) was decreased in the treatment group of CDDP and trigonelline combination compared to the control or CDDP treatment group. The nucleus (cyan fluorescence) also expressed an abnormal shape in the combination group, and this phenotype of nucleus may illustrate the cell death. The results showed that Nrf2 inhibition can increase the chemotherapeutic sensitivity and narrow the tumor size significantly. To further clarify the association between Nrf2 expression and the clinical outcome, TCGA breast cancer datasets were used. The data matrices were classified by ER (estrogen receptor), PR (progesterone receptor), and HER2 (human epidermal growth factor receptor 2) status. Relapse-free survival data showed that breast cancer patients with low Nrf2 expression had a lower incidence of relapse compare to those with high Nrf2 expression in the PR^+^/ER^+^ (p < 0.05) or TNBC (Triple negative breast cancer) groups. This analysis suggested that high Nrf2 expression can be a poor prognostic indicator in breast cancers. In conclusion, Nrf2 plays the key regulator in drug sensitivity of *in vitro* and *in vivo* models, and Nrf2 may be a potential target for treating drug resistance in breast tumors, especially under hypoxia microenvironment.

## DISCUSSION

**Figure 7:**
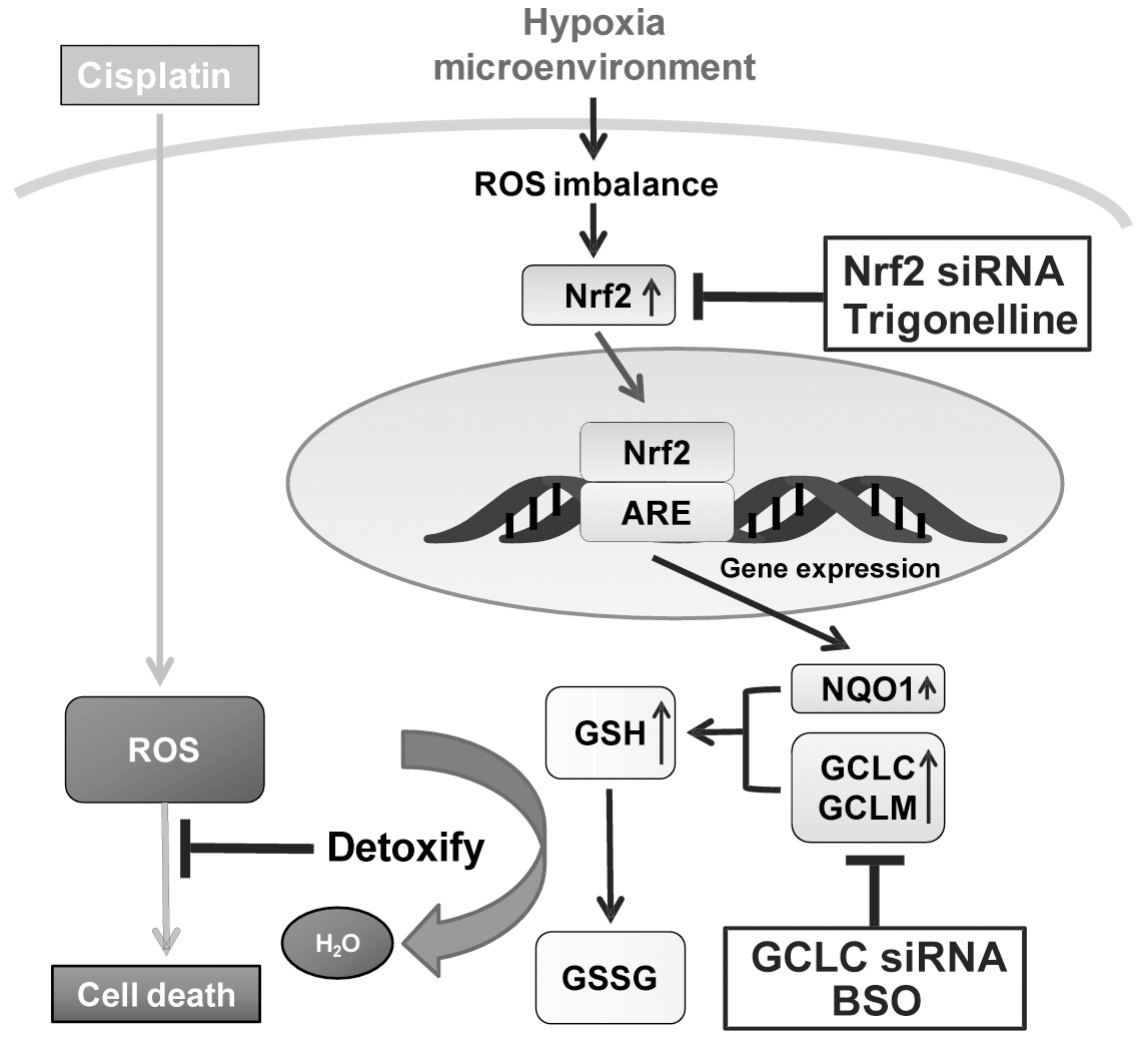
Model of Nrf2-induced drug resistance under hypoxia A schematic diagram of Nrf2-induced drug resistance under hypoxia and the modulation in MCF7 was shown. Nrf2 activity was evoked by the hypoxia-induced ROS imbalance, and the downstream GCLC enzyme was essential to increase the glutathione content to detoxify the cytotoxicity of CDDP. siNrf2, trigonelline, siGCLC, and BSO can block hypoxia-induced drug resistance, and may be useful as therapeutic agents combined with chemotherapeutic drugs.

The chemoresistance caused by hypoxia contribute significantly to these poor treatment outcomes. Therefore, understanding the key regulatory factors for hypoxia-induced chemoresistance is critical to improve cancer treatments. Nrf2 is regarded as a pro-tumorigenic factor in many tumor types by accelerating stress adaption, increasing drug resistance and driving oncogenesis [34–37]. Although the connection between Nrf2 and hypoxia-induced drug resistance remains unknown. In this study, we dissected the causal role of Nrf2 activation for the hypoxia-induced drug resistance in breast cancer cells. We have provided compelling experimental evidence by showing that hypoxia increases the drug resistance of MCF7 through Nrf2 activation. The Nrf2 activation was triggered by hypoxia-induced ROS, and the downstream GCLC enzyme was essential to increase the glutathione content to detoxify the cytotoxicity of CDDP. Therefore, the hypoxia-induced ROS-Nrf2-GCLC pathway increases the drug resistance and likely causes treatment failure in breast cancer cells. The underlying mechanism of hypoxia-induced drug resistance is schematically depicted in Figure 7.

In clinic, TNBC is considered to be a tumor types with strong drug resistance and poor response to treatment [38]. The TCGA data showed that the TNBC group has a lower survival rate compared with the ER^+^/PR^+^ group (Figure 6C, 6D). In both ER^+^/PR^+^ and TNBC group, patients with higher Nrf2 level got lower survival rate (Figure 6). CDDP is a common first-line chemotherapeutic drug for breast cancers, especially TNBC without target therapeutics [39]. Similarly, the inhibition of Nrf2 can significantly decrease CDDP resistance in both Nrf2-expressing MCF7 and MDAMB231 cells under hypoxia (Figure 5G, 5H).

Hif-1α expression has been associated with the chemo-resistance of cancer cells by inhibiting apoptosis and induction of drug efflux [40, 41]. Surprisingly, we found that Hif-1α may be not the major regulator in hypoxia-induced drug resistance in our model. Inhibition of Hif-1α by siRNA did not significantly decrease CDDP resistance under hypoxia (Figure 2E). In addition, Nrf2 increased the expression of antioxidant protein (GCLC/GCLM) and detoxification protein (MRP-1) (Figure 2B, Figure 3G). The results suggested that Nrf2 activity may be more important than Hif-1α in causing hypoxia–induced CDDP resistance through the ARE-mediated transcriptional regulation.

Previous studies have indicated hypoxia leads to intracellular oxidative imbalance [1]. This concept is supported by the highly dynamic ROS levels at different time points under hypoxia. In this study, Hypoxia-induced ROS at 4h leads to the activation of Nrf2, and decrease the ROS at 8 hours. At 8 hours, we also observed an increase in the nuclear translocation of Nrf2. At 24 hours, even though the nuclear translocation of Nrf2 is more dramatic than 8h (Figure 2A, 4D), this increased Nrf2 may not be able to cope with the hypoxia-induced ROS accumulation, thus resulting in another ROS surge. These data suggested that Nrf2 is upregulated by the ROS imbalance caused by hypoxia.

In summary, our study suggests the important role of the hypoxia-induced ROS-Nrf2-GCLC-GSH pathway in the hypoxia-induced chemoresistance. Targeting Nrf2 could be a potential therapy to decrease drug resistance and improve the treatment of solid breast tumors. Nrf2 activation can be a good marker for the selection of chemoresistant cells that most likely benefit from combined therapy with Nrf2 inhibitors to reduce hypoxia-induced drug resistance.

## MATERIALS AND METHODS

### Antibodies, chemicals, siRNA and plasmids

Primary antibodies specific for HIF-1α, Nrf2, p-Nrf2, GCLM, MRP-1, GAPDH, and Histone H3 were purchased from Genetex. The NQO1-specific antibody was purchased from Epitomics. The GCLC antibody was purchased from BTLAB. The rabbit secondary antibody conjugated with HRP and the rabbit secondary antibody conjugated with dylight 488 were purchased from Jackson. The following chemicals, including Thiazolyl Blue Tetrazolium Bromide (MTT), dimethyl sulfoxide (DMSO), cisplatin (CDDP), trigonelline, buthionine sulfoximine (BSO), dicoumarol (DIC), dithiothreitol (DTT), and N-acetyl-L-cysteine (NAC), were purchased from Sigma Aldrich. Neomycin (G418) was purchased from Merck. The following siRNAs were purchased from Omic Bio: HIF-1α (CCA UAU AGA GAU ACU CAA ATT), Nrf2 (GCA CCU UAU AUC UCG AAG UTT), GCLC (GCA UAG ACA CCA UCA ATT), and NQO1 (GUG GCU UCC AAG UCU UAG ATT). The PCDNA3-Myc3-Nrf2 plasmid was purchased from Addgene (Plasmid #21555).

### Cell culture

The human breast cancer cell lines MCF7 and MDAMB231 were purchased from American Type Culture Collection (ATCC), cultured in DMEM (Invitrogen) supplemented with 10% FBS (Gibco) and 1% penicillin/streptomycin (Invitrogen) in a 5% CO_2_ atmosphere at 37°C. MCF7-Nrf2 cells were established with stable Nrf2 overexpression and were selected by G418 (400 μg/ml) for up to 4 weeks. The Nrf2 protein level was measured by western blotting every week to ensure the expression of Nrf2.

### Cell viability analysis

Cells were cultured in 96-well plates and incubated for 24 hours. After treatments for the indicated times, 500 μg/ml MTT was added to cells for 2 hours. Then, the MTT crystal was dissolved by DMSO, and the OD absorbance was measured with an ELISA reader at 550 nm.

### Western blotting

Cells were lysed in RIPA buffer with protease inhibitor (sigma) and homogenized by sonicator (Branson), and proteins were separated by SDS-PAGE and transferred to nitrocellulose membranes. The membranes were blocked with 5% milk for 1 hour and incubated with targeted primary antibodies overnight at 4°C, and then incubated with HRP-conjugated secondary antibodies at room temperature for 1 hour. Finally, the signals were enhanced by ECL (Millipore) and detected with X-film exposure. Protein levels were quantified with Gel-Pro analyzer software and normalized to GAPDH.

### Total GSH measurement

GSH levels were measured with a GSH assay kit (Cayman, Ann Arbor, MI, USA). In brief, cells (2×10^5^) were incubated with indicated treatments, the cell pellets were collected with cold GSH MES buffer, homogenized by a sonicator, and then centrifuged at 10000 rpm for 15 minutes at 4°C. 50 μl of the supernatants were added to 150 μl Assay Cocktail (20 ml vial Assay Cocktail containing 11.25 ml MES Buffer, 0.45 ml Cofactor Mixture, 2.1 ml Enzyme Mixture, 2.3 ml water, and 0.45 ml DTNB), and the absorbances were measured at 405-414 nm with an ELISA reader at five-minute intervals for 30 minutes.

### Nuclear extraction

The nuclear extraction kit (Thermo Scientific) was used to purify the nuclear proteins. Cells (2×10^5^) were cultured in 6-well plates and incubated for the indicated treatments, the cell pellets were collected by trypsin-EDTA, vortexed intensely in 200 μl ice-cold CER I for 15 seconds, and incubated on ice for 10 minutes. Then, samples were washed in cold CER II, vortexed intensely for 5 seconds, and centrifuged at 12000 rpm for 5 minutes. The supernatants were collected as cytoplasmic extracts. The pellets were vortexed in 50 μl ice-cold NER for 15 seconds and then incubated on ice for 10 minutes and centrifuged at 12000 rpm for 10 minutes at 4°C. The supernatants were collected as nuclear extracts.

### Immunofluorescence

Cells were cultured on coverslips coated with 0.1% gelatin (Sigma) and incubated for the indicated treatments. Cells were fixed with 4% paraformaldehyde for 10 minutes, blocked with 10% FBS with 0.1% Triton-X 100 for 15 minutes, and incubated with targeted primary antibodies overnight at 4°C, then incubated with secondary antibodies at room temperature for 1 hour, and incubated with 0.4% DAPI for 10 minutes. The samples were mounted on the slides with mounting medium (Ibidi), and the images were captured by fluorescent microscopy (Leica).

### Luciferase reporter assay

The Dual-Glo luciferase assay kit (Promega) was used to perform the antioxidant response element (ARE) luciferase reporter assay. Cells were transfected with pARE-CMV (Addgene) plasmid containing a firefly luciferase gene and pRL-CMV (Promega) containing a Renilla luciferase plasmid. After the treatments, cells were collected with RIPA buffer (50 mM Tris-base, Ph 7.5, 150 mM NaCl, 1 mM EDTA, 0.1% SDS), the Dual-Glo luciferase assay reagent was added to the samples and incubated for 10 minutes at 25°C, and the firefly luminescence activities were measured (Berthold). Then, Dual-Glo Stop & Glo reagent was added to the samples and incubated for another 10 minutes at 25°C. The Renilla luminescence activities were measured in the luminometer, and normalized by the Renilla activities.

### ROS detection

Cells (2×10^5^) were incubated with 10 μM dichlorodihydrofluorescein diacetate (DCFHDA) (Molecule Probe) for 30 minutes at 37°C, and collected by trypsin-EDTA. The samples were analyzed by FACS Calibur flow cytometry with FL1-H parameters (BD Biosciences).

### Plasmid and siRNA transfection

Cells (2×10^5^) were cultured in each well of the 6-well plates and incubated for 24 hours, and the mixed reagent containing plasmid or siRNA with transfection reagent (Omic Bio) was added, which was previously incubated for 20 minutes, for 24 hours, and the levels of target proteins were examined by western blot.

### Animals

Male ICR mice (10 weeks old) were purchased from the National Taiwan University Animal Center. All mice were maintained under temperature- and humidity-controlled conditions and treated according to National Institutes of Health guidelines. All procedures were approved by the Laboratory Animal Committee of the College of Medicine, National Taiwan University.

### *In vivo* experiments

The *in vivo* tumor model in the ear was performed according to a previous study [42]. Four groups of experiments were performed, including treatment with PBS, CDDP, CDDP combined with trigonelline and trigonelline alone. MCF7 (1 × 10^7^) were subcutaneously injected in the center of the ears of the mice for 5 days. The ears were locally injected at the center of tumor masses with PBS, 2 μg/ml CDDP, CDDP combined with 2 μM trigonelline and trigonelline alone on days 5, 7, 9 and 11. The ears were photographed under stereomicroscopy, and the, the maximum length (horizontal on the ear plane), maximum width (vertical to the length on the ear plane), and the thickness (vertical to the ear plane) were measured by digital caliper on days 5, 7, 9 and 11, then tumor volumes were calculated by the formula “(4/3)×π×maximum length×maximum width× height”. The mice were sacrificed on day 11, and tumor status was photographed.

### TCGA database analysis

Clinical data were obtained from the TCGA open-access database. The breast cancer matrix dataset was downloaded from the breast invasive carcinoma [BRCA] database, including vital status, tumor status, overall survival, estrogen receptor (ER), progesterone receptor (PR), and human epidermal growth factor receptor 2 (HER2) statuses. The matrix dataset was classified into the ER/PR positive (ER^+^/PR^+^) group and the triple negative breast cancer (TNBC, ER^−^/PR^−^/HER2^−^) group according to tumor status. Two subgroups were further identified as high and low Nrf2 expression subgroups by the Nrf2 expression in both ER^+^/PR^+^ and TNBC groups. Tumors with Nrf2 level above the mean of all Nrf2 expression were identified as high Nrf2 expression, and others were grouped as low Nrf2 expression.

### Statistical analysis

All experiments were performed at least 3 times. Statistical significance between groups was determined by Student's t test and one-way ANOVA. The overall TCGA data were imported into GraphPad Prism (GraphPad Software, Inc.) and examined by the Mantel-Cox log-rank test. P values < 0.05 were considered statistically significant.

## SUPPLEMENTARY FIGURES



## References

[1] Bell EL, Klimova TA, Eisenbart J, Schumacker PT, Chandel NS (2007). Mitochondrial reactive oxygen species trigger hypoxia-inducible factor-dependent extension of the replicative life span during hypoxia. Mol Cell Biol.

[2] Chi JT, Wang Z, Nuyten DS, Rodriguez EH, Schaner ME, Salim A, Wang Y, Kristensen GB, Helland A, Borresen-Dale AL, Giaccia A, Longaker MT, Hastie T, Yang GP, van de Vijver MJ, Brown PO (2006). Gene expression programs in response to hypoxia: cell type specificity and prognostic significance in human cancers. PLoS Med.

[3] Dang CV (2012). Links between metabolism and cancer. Genes Dev.

[4] Gatza ML, Kung HN, Blackwell KL, Dewhirst MW, Marks JR, Chi JT (2011). Analysis of tumor environmental response and oncogenic pathway activation identifies distinct basal and luminal features in HER2-related breast tumor subtypes. Breast Cancer Res.

[5] Tang X, Lucas JE, Chen JL, LaMonte G, Wu J, Wang MC, Koumenis C, Chi JT (2012). Functional interaction between responses to lactic acidosis and hypoxia regulates genomic transcriptional outputs. Cancer Res.

[6] Postovit LM, Adams MA, Lash GE, Heaton JP, Graham CH (2002). Oxygen-mediated regulation of tumor cell invasiveness. Involvement of a nitric oxide signaling pathway. J Biol Chem.

[7] Frederiksen LJ, Siemens DR, Heaton JP, Maxwell LR, Adams MA, Graham CH (2003). Hypoxia induced resistance to doxorubicin in prostate cancer cells is inhibited by low concentrations of glyceryl trinitrate. J Urol.

[8] Teicher BA (1994). Hypoxia and drug resistance. Cancer Metastasis Rev.

[9] Li C, Lu HJ, Na FF, Deng L, Xue JX, Wang JW, Wang YQ, Li QL, Lu Y (2013). Prognostic role of hypoxic inducible factor expression in non-small cell lung cancer: a meta-analysis. Asian Pac J Cancer Prev.

[10] Zheng SS, Chen XH, Yin X, Zhang BH (2013). Prognostic significance of HIF-1alpha expression in hepatocellular carcinoma: a meta-analysis. PLoS One.

[11] Song X, Liu X, Chi W, Liu Y, Wei L, Wang X, Yu J (2006). Hypoxia-induced resistance to cisplatin and doxorubicin in non-small cell lung cancer is inhibited by silencing of HIF-1alpha gene. Cancer Chemother Pharmacol.

[12] Ogiso Y, Tomida A, Lei S, Omura S, Tsuruo T (2000). Proteasome inhibition circumvents solid tumor resistance to topoisomerase II-directed drugs. Cancer Res.

[13] Sullivan R, Pare GC, Frederiksen LJ, Semenza GL, Graham CH (2008). Hypoxia-induced resistance to anticancer drugs is associated with decreased senescence and requires hypoxia-inducible factor-1 activity. Mol Cancer Ther.

[14] Friesen C, Kiess Y, Debatin KM (2004). A critical role of glutathione in determining apoptosis sensitivity and resistance in leukemia cells. Cell Death Differ.

[15] McLellan LI, Wolf CR (1999). Glutathione and glutathione-dependent enzymes in cancer drug resistance. Drug Resist Updat.

[16] Townsend DM, Tew KD (2003). The role of glutathione-S-transferase in anti-cancer drug resistance. Oncogene.

[17] Wang XJ, Hayes JD, Wolf CR (2006). Generation of a stable antioxidant response element-driven reporter gene cell line and its use to show redox-dependent activation of nrf2 by cancer chemotherapeutic agents. Cancer Res.

[18] Marengo B, De Ciucis C, Verzola D, Pistoia V, Raffaghello L, Patriarca S, Balbis E, Traverso N, Cottalasso D, Pronzato MA, Marinari UM, Domenicotti C (2008). Mechanisms of BSO (L-buthionine-S,R-sulfoximine)-induced cytotoxic effects in neuroblastoma. Free Radic Biol Med.

[19] Palomares T, Carames M, Garcia-Alonso I, Alonso-Varona A (2009). Glutathione modulation reverses the growth-promoting effect of growth factors, improving the 5-fluorouracil antitumour response in WiDr colon cancer cells. Anticancer Res.

[20] Kolamunne RT, Dias IH, Vernallis AB, Grant MM, Griffiths HR (2013). Nrf2 activation supports cell survival during hypoxia and hypoxia/reoxygenation in cardiomyoblasts; the roles of reactive oxygen and nitrogen species. Redox Biol.

[21] Jaiswal AK (2000). Regulation of genes encoding NAD(P)H:quinone oxidoreductases. Free Radic Biol Med.

[22] Solis WA, Dalton TP, Dieter MZ, Freshwater S, Harrer JM, He L, Shertzer HG, Nebert DW (2002). Glutamate-cysteine ligase modifier subunit: mouse Gclm gene structure and regulation by agents that cause oxidative stress. Biochem Pharmacol.

[23] Tang X, Wang H, Fan L, Wu X, Xin A, Ren H, Wang XJ (2011). Luteolin inhibits Nrf2 leading to negative regulation of the Nrf2/ARE pathway and sensitization of human lung carcinoma A549 cells to therapeutic drugs. Free Radic Biol Med.

[24] Chandel NS, McClintock DS, Feliciano CE, Wood TM, Melendez JA, Rodriguez AM, Schumacker PT (2000). Reactive oxygen species generated at mitochondrial complex III stabilize hypoxia-inducible factor-1alpha during hypoxia: a mechanism of O2 sensing. J Biol Chem.

[25] Guzy RD, Hoyos B, Robin E, Chen H, Liu L, Mansfield KD, Simon MC, Hammerling U, Schumacker PT (2005). Mitochondrial complex III is required for hypoxia-induced ROS production and cellular oxygen sensing. Cell Metab.

[26] Jiang H, Xie J, Xu G, Su Y, Li J, Zhu M, Wang M (2013). Hypoxia regulates reactive oxygen species levels in SHG-44 glioma cells. Neural Regen Res.

[27] Reczek CR, Chandel NS (2014). ROS-dependent signal transduction. Curr Opin Cell Biol.

[28] Hagen T (2012). Oxygen versus Reactive Oxygen in the Regulation of HIF-1alpha: The Balance Tips. Biochem Res Int.

[29] Dasari S, Tchounwou PB (2014). Cisplatin in cancer therapy: molecular mechanisms of action. Eur J Pharmacol.

[30] Arlt A, Sebens S, Krebs S, Geismann C, Grossmann M, Kruse ML, Schreiber S, Schafer H (2013). Inhibition of the Nrf2 transcription factor by the alkaloid trigonelline renders pancreatic cancer cells more susceptible to apoptosis through decreased proteasomal gene expression and proteasome activity. Oncogene.

[31] Wind NS, Holen I (2011). Multidrug resistance in breast cancer: from in vitro models to clinical studies. Int J Breast Cancer.

[32] Buelna-Chontal M, Zazueta C (2013). Redox activation of Nrf2 & NF-kappaB: a double end sword?. Cell Signal.

[33] Apopa PL, He X, Ma Q (2008). Phosphorylation of Nrf2 in the transcription activation domain by casein kinase 2 (CK2) is critical for the nuclear translocation and transcription activation function of Nrf2 in IMR-32 neuroblastoma cells. J Biochem Mol Toxicol.

[34] Geismann C, Arlt A, Sebens S, Schafer H (2014). Cytoprotection “gone astray”: Nrf2 and its role in cancer. Onco Targets Ther.

[35] Jaramillo MC, Zhang DD (2013). The emerging role of the Nrf2-Keap1 signaling pathway in cancer. Genes Dev.

[36] Shelton P, Jaiswal AK (2013). The transcription factor NF-E2-related factor 2 (Nrf2): a protooncogene?. FASEB J.

[37] Sporn MB, Liby KT (2012). NRF2 and cancer: the good, the bad and the importance of context. Nat Rev Cancer.

[38] Griffiths CL, Olin JL (2012). Triple negative breast cancer: a brief review of its characteristics and treatment options. J Pharm Pract.

[39] Sledge GW, Loehrer PJ, Roth BJ, Einhorn LH (1988). Cisplatin as first-line therapy for metastatic breast cancer. J Clin Oncol.

[40] Kim JW, Tchernyshyov I, Semenza GL, Dang CV (2006). HIF-1-mediated expression of pyruvate dehydrogenase kinase: a metabolic switch required for cellular adaptation to hypoxia. Cell Metab.

[41] Warfel NA, El-Deiry WS (2014). HIF-1 signaling in drug resistance to chemotherapy. Curr Med Chem.

[42] Yang SH, Wang SM, Syu JP, Chen Y, Wang SD, Peng YS, Kuo MF, Kung HN (2014). Andrographolide induces apoptosis of C6 glioma cells via the ERK-p53-caspase 7-PARP pathway. Biomed Res Int.

