# Supplementary material for: Nrf2 is the key to chemotherapy resistance in MCF7 breast cancer cells under hypoxia
Source: Oncotarget. 2016 Feb 15;7(12):14659–72. doi: 10.18632/oncotarget.7406 (PMC4924742; doi:10.18632/oncotarget.7406)
Supplement: Supplementary file 1 [file oncotarget-07-14659-s001.pdf]

SUPPLEMENTARY FIGURES

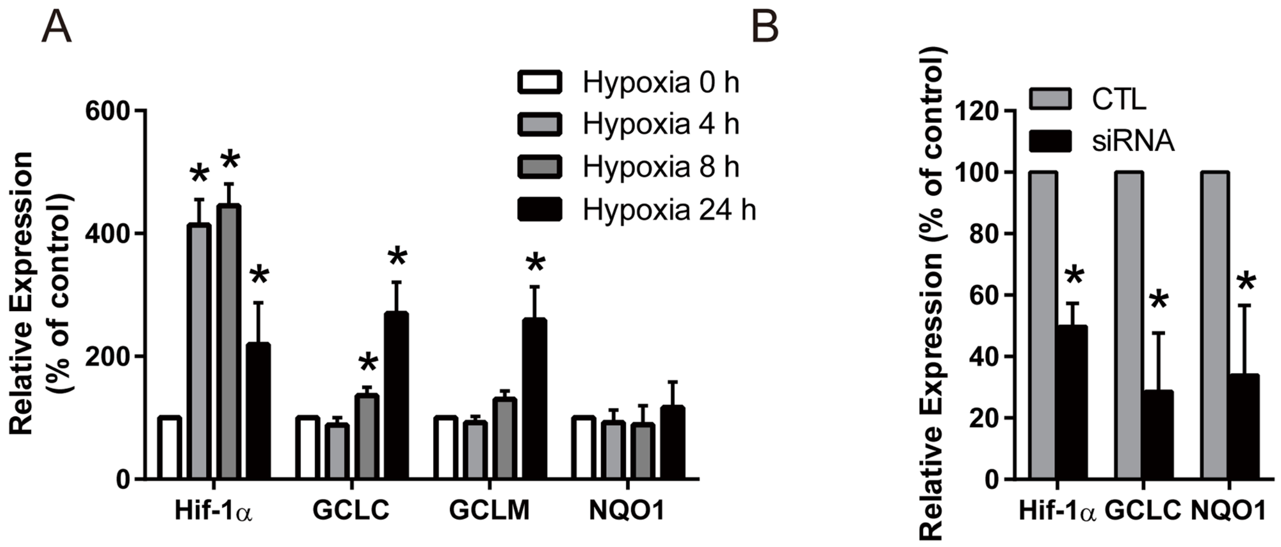

Supplementary Figure 1. A. The protein levels of Figure 2B were quantified and normalized with GAPDH. Results are means ±S.D. for three independent experiments. N=3, *, P<0.05 compared with the 0 hour group. **B.** The protein levels of Figure 2D were quantified and normalized with GAPDH. Results are means ±S.D. for three independent experiments. N=3, *, P<0.05 compared with the siCTL group.

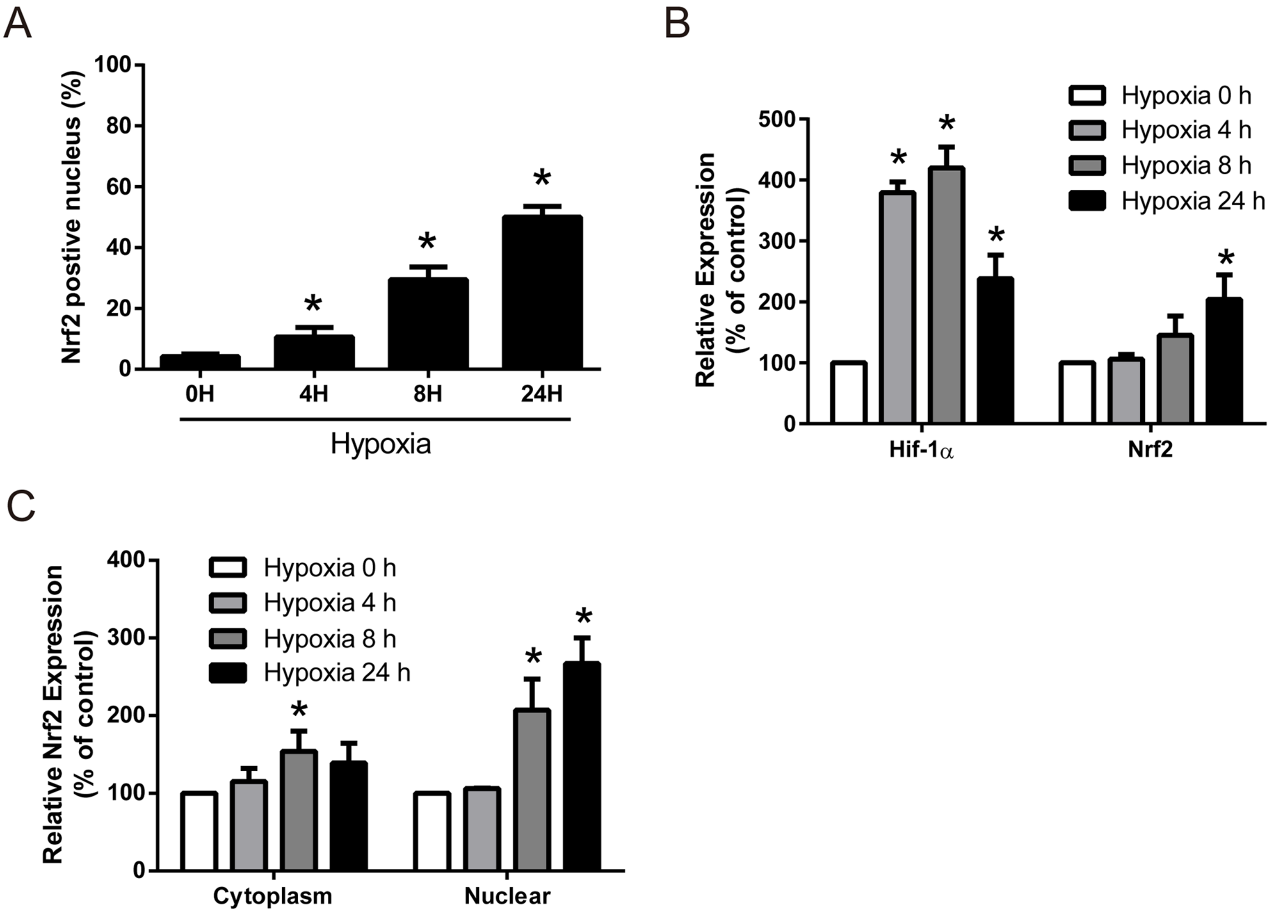

Supplementary Figure 2. A. The cells with Nrf2 positive nucleus of Figure 3A were quantified. Results are showed by means ±S.D. for three independent experiments. N=3, *, P<0.05 compared with the 0 hour group. **B.** The protein levels of Figure 3B were quantified and normalized with GAPDH. Results are means ±S.D. for three independent experiments. N=3, *, P<0.05 compared with the 0 hour group. **C.** The protein levels of Figure 3C were quantified and normalized with cytoplasm control (GAPDH) and nuclear control (Histone H3). Results are means ±S.D. for three independent experiments. N=3, *, P<0.05 compared with the 0 hour group.

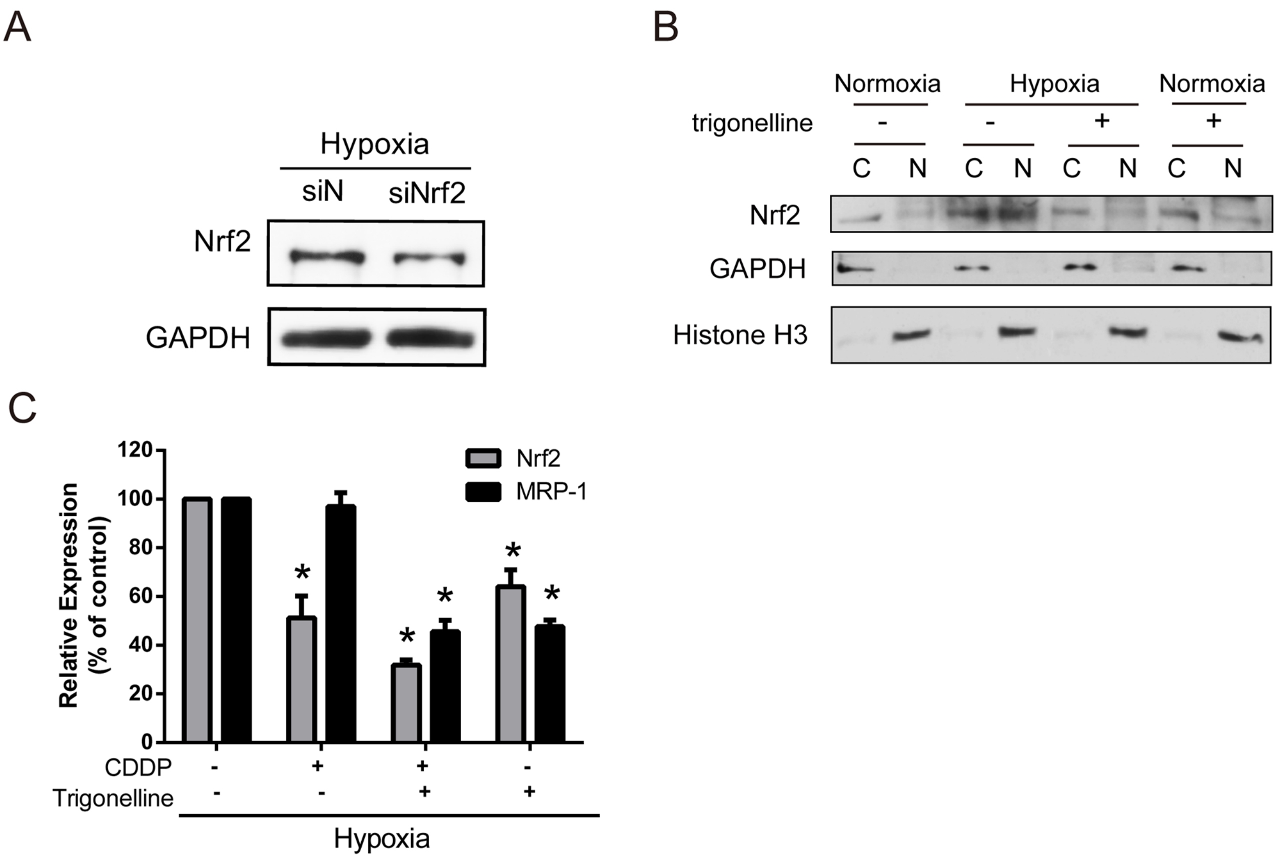

Supplementary Figure 3. A. MCF7 cells were treated with negative siRNA (siNeg), Nrf2 siRNA, for 24 hours under hypoxia, and the inhibition efficiency of Nrf2 protein level was detected by western blotting. **B.** MCF7 cells were treated with 1 μg/ml CDDP combined with or without 3 hours pretreatment of trigonelline (Nrf2 inhibitor), or 1 μM trigonelline alone under hypoxia for 24 hours. Cytosolic (C)/ nuclear (N) proteins were extracted. The protein levels Nrf2, GAPDH, Histone H3 were detected by western blot. GAPDH is the loading control for cytosolic fraction, and Histone H3 is the loading control for nuclear fraction. **C.** The protein levels of Figure 3G were quantified and normalized with GAPDH. Results are means ±S.D. for three independent experiments. N=3, *, P<0.05 compared with the control group.

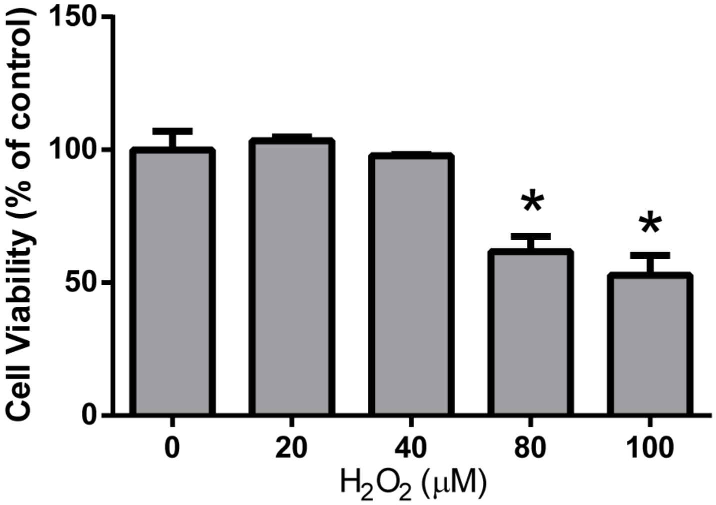

Supplementary Figure 4. MCF7 cells were treated with 40 μM H2O2 for 24 hours. The cell viability was detected by MTT assay. N=3, *, P<0.05 compared with the control groups.

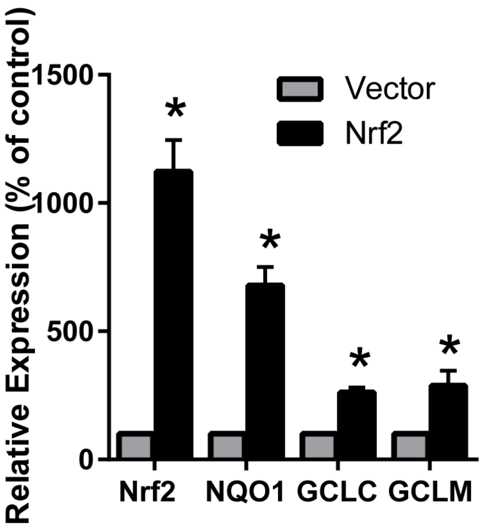

Supplementary Figure 5. The protein levels of Figure 5A were quantified and normalized with GAPDH. Results are means ±S.D. for three independent experiments. N=3, *, P<0.05 compared with the vector control group.

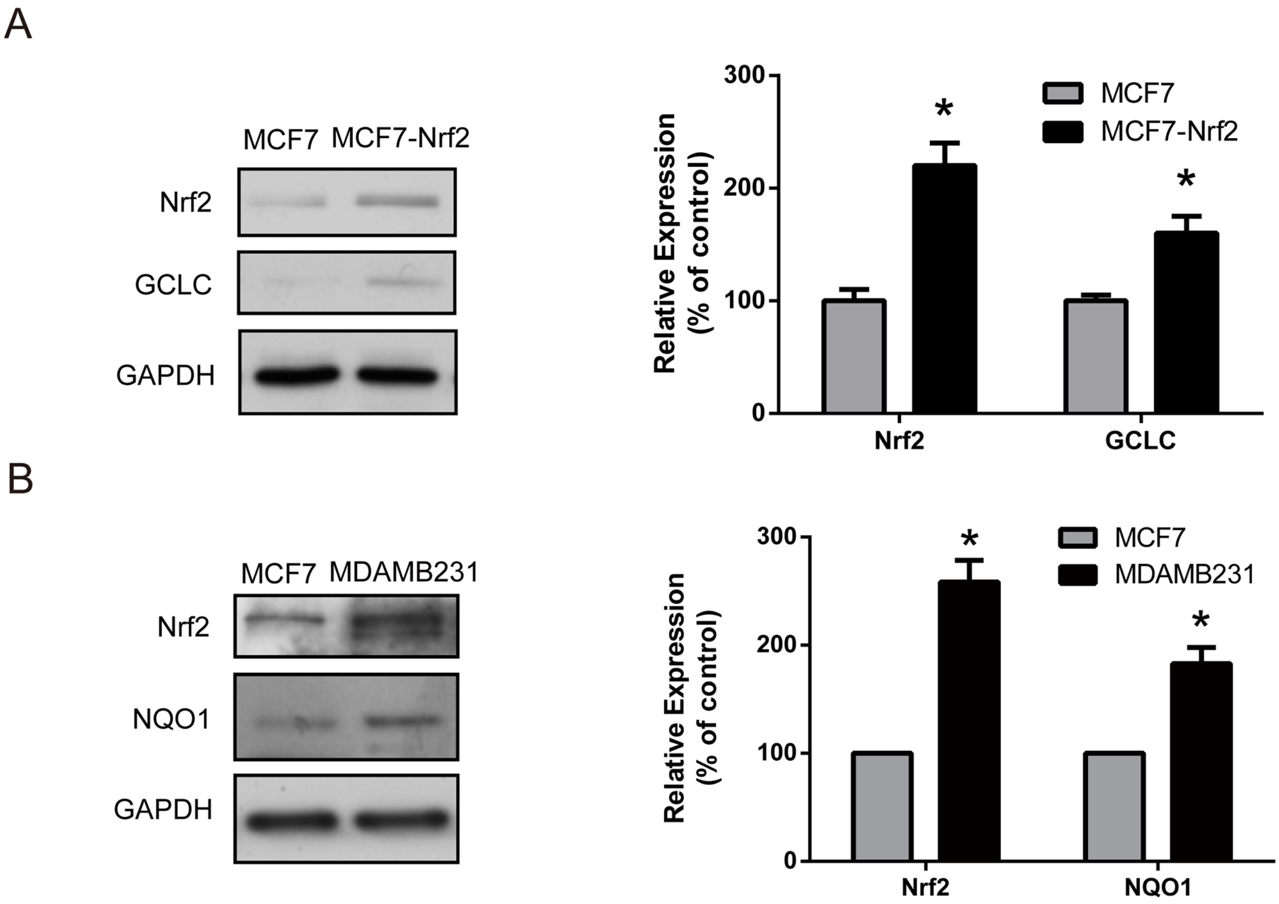

Supplementary Figure 6. A. The proteins levels of MCF7 and MCF7-Nrf2 cells were detected by western blot with anti-Nrf2, GCLC and GAPDH. Results are means ±S.D. for three independent experiments. N=3, *, P<0.05 compared with the MCF7 group. **B.** The proteins levels of MCF7 and MDAMB231 cells were detected by western blot with anti-Nrf2 and GAPDH. Results are means ±S.D. for three independent experiments. N=3, *, P<0.05 compared with the MCF7 group.

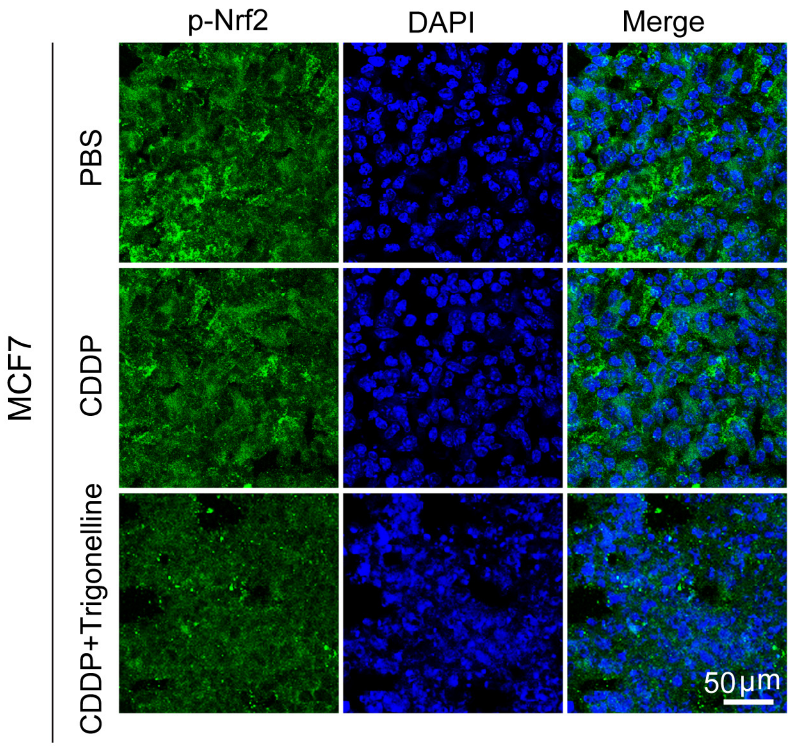

Supplementary Figure 7. The mice were sacrificed on day 11, and the Nrf2 activity was detected by IHC method with p-Nrf2 antibody. The p-Nrf2 antibody (green fluorescence), and nucleus (blue fluorescence) were stained. The pictures were photographed with a SP5 confocal microscopy.
